# Illness Perceptions and Fear of Recurrence Among Myocardial Infarction Survivors: The Mediating Role of Psychological Flexibility

**DOI:** 10.31083/AP44019

**Published:** 2025-06-19

**Authors:** Yan Wang, Qingyan Tian, Junhui Yan, Xi Chen, Fen Xiong, Haocheng Yang, Zhihui Huang, Hongjuan Wen, Botang Guo, Ping Tang

**Affiliations:** ^1^Department of Cardiology, First Affiliated Hospital of PLA Air Force Military Medical University, 710032 Xi’an, Shaanxi, China; ^2^Department of General Practice, The Affiliated Luohu Hospital of Shenzhen University Medical School, 518001 Shenzhen, Guangdong, China; ^3^Department of Nursing, The Affiliated Luohu Hospital of Shenzhen University Medical School, 518001 Shenzhen, Guangdong, China; ^4^College of Health Management, Changchun University of Traditional Chinese Medicine, 130117 Changchun, Jilin, China

**Keywords:** myocardial infarction, recurrence, illness perception, psychological flexibility

## Abstract

**Background:**

Myocardial infarction (MI) patients often experience fear of recurrence, which affects their psychological well-being and quality of life. This study aimed to explore the relationship between illness perception, psychological flexibility, and fear of recurrence, as well as to investigate the demographic factors associated with fear of recurrence in MI patients.

**Methods:**

A cross-sectional survey was conducted, enrolling 466 MI patients to complete questionnaires assessing general information, disease-related factors, illness perception, psychological flexibility, and fear of recurrence. Correlation analysis, analysis of variance, and mediation effect analysis were used to explore the relationships between these factors and fear of recurrence.

**Results:**

Gender, monthly income, marital status, alcohol consumption, New York Heart Association functional classification (NYHA classification), and number of chronic diseases were significantly associated with fear of recurrence. Illness perception was positively correlated with patients’ fear of recurrence. Psychological flexibility was negatively correlated with fear of recurrence and played a mediating role between illness perception and fear of recurrence, mediating the negative impact of illness perception on fear.

**Conclusion:**

The findings of this study emphasize the critical role of psychological flexibility in mitigating the fear of recurrence among myocardial infarction survivors. By targeting modifiable factors such as psychological flexibility and illness perceptions, healthcare providers can develop more effective interventions aimed at improving mental health and overall quality of life for these patients.

## Main Points

1. **Illness perception and clinical interventions**: Negative illness perceptions significantly increase fear of recurrence in myocardial infarction (MI) survivors, highlighting the need for targeted interventions, such as psychological flexibility training, to improve mental health outcomes.

2.** Psychological flexibility acts as a protective factor**: Higher levels of psychological flexibility are associated with lower levels of fear of recurrence, suggesting its critical role in helping patients manage their fears more effectively.

3. **Mediation effect of psychological flexibility**: Psychological flexibility mediates the relationship between illness perception and fear of recurrence, accounting for 35.1% of the total effect, demonstrating its substantial impact on reducing fear of recurrence.

4. **Demographic factors inform personalized interventions**: Gender, income, marital status, alcohol consumption, and the presence of chronic diseases significantly influence fear of recurrence, underscoring the need for tailored clinical approaches to address these factors in MI survivors.

## 1. Introduction

Myocardial infarction (MI) is a severe cardiovascular disease that profoundly impacts the physical and mental well-being of patients. Despite advances in medical technology improving the survival rate of MI patients, survivors often face numerous mental health challenges during the recovery process. MI survivors frequently report significant psychological distress, with depression and anxiety being the most common. Studies have found that the prevalence of depression and anxiety after MI is respectively as high as 28.7% [1] and 32% [2]. These psychological issues increase the burden on the cardiovascular system and elevate the risk of recurrent cardiac events [3]. Such studies indicate that MI survivors face multiple mental health challenges that require serious attention and intervention.

In addition to depression and anxiety, fear of recurrence (FoR) is another significant psychological challenge faced by MI survivors. FoR refers to the excessive worry and fear in patients about the recurrence of the disease, which is a common psychological stress response [4]. Research has found that up to 59% of coronary heart disease (CHD) patients report moderate to severe levels of FoR [5]. FoR can trigger panic attacks and avoidance behaviors, severely impacting patient mental health and quality of life [6]. A longitudinal study revealed that higher levels of FoR were associated with lower quality of life among MI survivors [7]. Consequently, FoR has become a crucial issue in the research on the mental health of MI survivors.

Given the significant impact of psychological problems on the recovery of MI survivors, researchers have begun to investigate the factors influencing post-MI mental health. Some studies have found that demographic factors (such as age, gender, education level), clinical factors (such as MI severity, complications) and social support may all influence the mental health of MI survivors [8, 9]. However, most of these factors are difficult to change; therefore, researchers have started to focus on modifiable psychological factors, such as illness perceptions and psychological flexibility [10, 11]. Despite this, existing research still lacks a comprehensive integration of factors such as illness perception and psychological flexibility to explain the mental health of MI survivors.

This study aims to develop a psychological model that explains the fear of recurrence in MI survivors from the perspective of illness perception and psychological flexibility. The proposed model seeks to provide a more comprehensive understanding of the psychological factors contributing to FoR among MI survivors, which may inform the development of targeted interventions to promote their mental well-being and overall recovery outcomes. By investigating the relationships between illness perception, psychological flexibility and FoR, this research endeavors to bridge the gaps in the current literature and offer insights for healthcare professionals working with MI survivors

### 1.1 Relationship Between Illness Perception and Fear of Recurrence

Illness perception refers to an individual’s subjective cognition and understanding of their disease, including their views on the causes, consequences, duration and controllability of an illness [12]. The self-regulatory model proposed by Leventhal *et al*. [13] serves as an important theoretical foundation for research on illness perception [14]. This model posits that when individuals face an illness, they form cognitive representations of the disease and adopt corresponding coping strategies based on these representations, which in turn influence their emotional and behavioral responses. The model emphasizes the central role of illness perception in an individual’s adaptation to a disease process and has been widely applied in research on the mental health of chronic disease patients.

Numerous studies have demonstrated that illness perception is closely related to the psychological adaptation of MI survivors. For example, Broadbent *et al*. [15] found that the illness perceptions during hospitalization of MI patients could predict their level of depression and anxiety following discharge. Another study discovered that patients who held negative illness perceptions (e.g., believed a disease had severe consequences, thus lacked a sense of control) after MI were more likely to develop symptoms of depression and post-traumatic stress disorder [16]. Previous studies, such as Xu *et al*. [17], have demonstrated similar relationships between illness perception and fear of recurrence in cancer patients, specifically breast cancer survivors. Their findings showed negative illness perceptions significantly increase fear of disease recurrence, a pattern that may extend to other chronic illness populations, including MI survivors. Such evidence further supports the notion that illness perception is a crucial factor affecting the psychological adaptation MI survivors and should be given due attention. Furthermore, illness perception may also influence treatment adherence and health behaviors for MI survivors [18]. Such findings suggest that illness perception is a crucial factor affecting psychological adaptation in MI survivors and should be given due attention.

Although the relationship between illness perception and the mental health of MI survivors has been extensively studied, evidence regarding the association between illness perception and fear of recurrence remains limited. A qualitative study revealed that the illness perceptions of MI survivors, particularly their thoughts about the consequences of the disease and the likelihood of recurrence, may influence their level of fear of recurrence [19]. Therefore, the aim here was to further empirically investigate through research the predictive role of the illness perceptions of MI survivors in their fear of recurrence.

### 1.2 Relationship Between Psychological Flexibility and Fear of Recurrence

Psychological flexibility is a concept originating from acceptance and commitment therapy (ACT), which refers to an individual’s ability to change or maintain behavior to achieve valuable goals based on the situational context [20]. ACT theory posits that psychological flexibility encompasses six core processes: acceptance, cognitive defusion, present moment awareness, self-as-context, values and committed action [21]. These processes are interconnected and jointly promote an individual’s ability to face internal experiences with an open and compassionate attitude and take actions that align with their personal values. Psychological flexibility is considered the core mechanism of ACT and plays a crucial role in individually promoting a person’s mental health [22].

Recently, psychological flexibility has received increasing attention in research into the mental health of chronic disease patients. For example, among chronic pain patients, higher levels of psychological flexibility are associated with lower pain sensitivity and less severe symptoms of depression and anxiety [23]. In patients with type 2 diabetes, psychological flexibility mediates the impact of diabetes symptoms on depression [24]. Moreover, ACT interventions targeting chronic disease patients have shown positive effects on improving depression, anxiety and quality of life by enhancing psychological flexibility [25]. This evidence from other chronic disease populations suggest that psychological flexibility may form an important protective factor for the mental health of chronic disease patients.

Although the role of psychological flexibility in chronic disease mental health is becoming increasingly prominent, research on this specific construct among MI survivors remains scarce. Spatola *et al*. [26] found that the psychological flexibility of MI patients was positively correlated with their post-traumatic growth and negatively correlated with post-traumatic stress disorder symptoms. This suggests that psychological flexibility may be a resilience factor for MI survivors. However, the study did not address the relationship between psychological flexibility and other mental health indicators, such as fear of recurrence. Given that ACT has been preliminarily applied to psychological interventions for cardiovascular disease patients with certain positive outcomes [27], an in-depth exploration of the mechanisms of psychological flexibility in this population is of importance for the development of targeted psychological interventions.

This study aims to examine the protective role of psychological flexibility among MI survivors, with the hope of enriching the research on factors influencing this population’s mental health. Here, it is hypothesized that MI survivors with higher levels of psychological flexibility may exhibit lower levels of fear of recurrence. Psychological flexibility may help MI survivors to accept their internal experiences related to the disease, detach from maladaptive thoughts about recurrence, focus on the present moment, clarify their values and engage in committed actions towards a meaningful life. By investigating the relationship between psychological flexibility and fear of recurrence, this research seeks to provide a new perspective on the psychological resilience factors of MI survivors and offer valuable insights for designing ACT-based interventions to alleviate their fear of recurrence and improve their overall mental well-being.

### 1.3 Mediating Role of Psychological Flexibility

Illness perception, as an individual’s subjective thoughts and understanding of their disease, may have a significant impact on psychological flexibility. According to ACT theory, an individual’s open attitude towards inner experiences (including disease-related thoughts and feelings) is an important manifestation of psychological flexibility [28]. When individuals hold negative perceptions of their illness, they often devote excessive attention to and avoidance of the disease, which may hinder them from viewing their inner experiences in an open and accepting manner, thus reducing their level of psychological flexibility. Furthermore, feelings of helplessness and uncontrollability in illness perception may also weaken an individual’s willingness and ability to take actions based on their values, further impacting psychological flexibility [29]. Such theoretical analyses suggest that illness perception may be an important influencing factor of psychological flexibility among MI survivors.

Given that illness perception may affect psychological flexibility and psychological flexibility is closely related to mental health, it is hypothesized here that psychological flexibility may play a mediating role between illness perception and fear of recurrence. Specifically, negative illness perceptions may increase fear of recurrence for MI survivors by reducing their psychological flexibility. This hypothesis aligns with ACT theory’s view on the mediating role of psychological flexibility between stress and mental health [20]. Although there is currently no direct evidence supporting this mediation model in MI survivors, research from other chronic disease populations provides preliminary support for this hypothesis. For example, Graham *et al*. [30] found that in patients with rheumatoid arthritis, illness perception affected the depression levels of patients through psychological flexibility. This suggests that psychological flexibility may be an important bridge connecting illness perception and mental health.

Constructing a mediation model of illness perception, psychological flexibility and fear of recurrence has important implications for research and practice on the mental health of MI survivors. This model helps to theoretically clarify the formative mechanisms of fear of recurrence among MI survivors, highlights the central role of psychological flexibility and enriches the understanding of factors influencing this population’s mental health. Practically, this model can provide new ideas for the development and optimization of psychological interventions for MI survivors. If psychological flexibility is confirmed as a mediator between illness perception and fear of recurrence, future interventions could focus on enhancing the psychological flexibility of patients for mediating the adverse impact of illness perception on fear of recurrence. Given ACT’s advantages in improving psychological flexibility [31], incorporating ACT elements into psychological interventions for MI survivors may help to enhance intervention effectiveness.

In summary, constructing and validating this mediation model will provide important insights for understanding and improving the mental health of MI survivors. By elucidating the relationships among illness perception, psychological flexibility and fear of recurrence, this research may contribute to the development of targeted interventions that promote psychological resilience and well-being in this vulnerable population. Future studies should aim to empirically test this model and explore the potential of ACT-based interventions in addressing the mental health needs of MI survivors.

### 1.4 Current Study

The present study aims to investigate the relationships among illness perception, psychological flexibility and fear of recurrence in a population of MI survivors, with a particular focus on examining whether psychological flexibility plays a mediating role between illness perception and fear of recurrence. By constructing and validating a mediation model, this study seeks to reveal the formation mechanism of fear of recurrence among MI survivors and provide a new perspective for research and intervention practices targeting the mental health of this population.

Based on the aforementioned theoretical analysis and literature review, this study proposes the following hypotheses:

**H_1_**: Illness perception has a significant positive correlation with fear of recurrence.

**H_2_**: Illness perception has a significant negatively correlated with psychological flexibility.

**H_3_**: Psychological flexibility is significantly negative correlation with fear of recurrence.

**H_4_**: Psychological flexibility mediates the relationship between illness perception and fear of recurrence.

## 2. Methods

### 2.1 Study Participants

The study participants were included MI patients from three tertiary grade A hospitals in Shaanxi Province, Guangdong Province and Jilin Province, China and data were collected between July and December 2023. All patients met the diagnostic criteria for MI and had entered the rehabilitation phase after receiving relevant treatment. Inclusion criteria: ① Age ≥18 years; ② Diagnosed with MI and had received treatment; ③ Able to independently understand and complete the questionnaires or do so under the guidance of researchers; ④ Agreed to participate in the study and signed an informed consent form. Exclusion criteria: ① Presence of severe mental disorders or cognitive impairments; ② Experienced major psychological stressors (such as the death of a family member or significant financial loss) in the recent past (within the last three months); ③ Presence of other severe chronic diseases (such as advanced cancer, end-stage renal disease) with a shorter life expectancy.

### 2.2 Sample Size Calculation

Using the sample size calculation formula: *n* = *Zp*^2^(1-*p*)/*d*^2^, where *Z* is the Z-value for the desired confidence level (1.96 for 95% confidence interval (CI)), *p* is the expected proportion of fear of recurrence in heart disease patients (estimated to be 0.53 [32]) and *d* is the desired precision (set at 0.05). Calculation showed a minimum of 387 participants were required and assuming a 10% non-response rate, the final sample size was set to 426 subjects.

### 2.3 Measurements

#### 2.3.1 Demographic Form

Included: patient age, gender, place of residence, marital status, education level, monthly income, smoking history, alcohol consumption history, New York Heart Association functional classification (NYHA classification), type of heart attack and medical history.

#### 2.3.2 Fear of Recurrence 

The Fear of recurrence in this study was measured using the 43-item Fear of Progression Questionnaire (FoP-Q), developed by Herschbach *et al*. [33], which has been widely used to assess disease-related fears in patients with chronic conditions. This scale consists of 43 items, with Likert scoring on a five point scale: 1. Represents never, 5. Represents always. The higher the total score, the stronger a patient’s fear of recurrence. This scale has been widely used in cancer and other patient populations, with good reliability and validity [5, 34]. The Cronbach’s alpha coefficient of this scale was 0.892 in this study.

#### 2.3.3 Psychological Flexibility 

The Psychological Flexibility Questionnaire (PFQ), developed by Benitzhak *et al*. [35], is a 20-item self-report measure that assesses five distinct domains of psychological flexibility. These domains include: positive perception of change (PPC: five items), self-characterization as flexible (SAF: five items), self-characterization as open and innovative (SOI: three items), perception of reality as dynamic and changing (RDC: four items) and perception of reality as multifaceted (RMF: three items). Respondents rate each item on a six-point Likert scale and the scores are then aggregated to yield a total PF score, with higher scores indicating greater levels of psychological flexibility. The PFQ has demonstrated strong psychometric properties in previous research, with Zukerman *et al*. [36] reporting a high reliability (Cronbach’s alpha = 0.740), with the equivalent Cronbach’s alpha coefficient in this study determined to be 0.857.

#### 2.3.4 Illness Perception

The Brief Illness Perception Questionnaire (BIPQ), developed by Broadbent *et al*. [37], comprised nine items, with the first eight items rated on a ten-point Likert scale ranging from 0 (not at all) to 10 (severely affected my life). These eight items cover various dimensions of illness perception, including: consequences, timeline, personal control, treatment control, identity, concern, understanding and emotional response. The final item of the BIPQ was an open-ended question that prompts respondents to list up to three factors they believe contributed to the development of their illness. The BIPQ allows for the calculation of an overall score, ranging from 0 to 80, which indicates the extent to which the illness is perceived as benign or threatening. Higher total scores suggest that the individual views their illness as more harmful and threatening. The scale has been widely studied in Chinese populations, with good reliability and validity [38, 39]. The Cronbach’s alpha coefficient of this scale was 0.926 in this study.

### 2.4 Data Collection and Quality Control

This study employed a structured questionnaire survey method, with trained researchers distributing and collecting questionnaires during follow-up visits after patient discharge. To ensure data validity and accuracy, researchers provided on-site guidance to patients during questionnaire completion to ensuring each question was correctly understood. During data collection, researchers checked the completeness and validity of each questionnaire to ensure that no data was missing or responses were invalid. If questionnaires were not correctly completed, researchers promptly contacted patients to supplement or refill the appropriate information. To maintain quality control, several measures were implemented. First, the team at each center consisted of researchers who had undergone uniform training to ensure consistency in survey methods and data collection standards. Second, for the data entry process, a double-entry and verification mechanism was adopted to ensure data entry accuracy. Third, regular data cleaning was performed to eliminate obvious outliers or unreasonable data points. In total, 500 questionnaires were distributed in this study and 466 valid questionnaires were ultimately collected, resulting in a response rate of 93.2%.

### 2.5 Statistical Analysis

Data analysis was performed using SPSS 26.0 software (IBM Corp., Armonk, NY, USA). Descriptive statistics were determined for general demographic characteristics, fear of recurrence scores, psychological flexibility scores and illness perception scores. Quantitative variables were expressed as mean ± standard deviation, while categorical variables were presented as frequencies and percentages. Independent samples *t*-tests or analysis of variance were employed to examine differences in fear of recurrence scores. Relationships between variables were examined via the Pearson correlation coefficient. PROCESS Model 4 was utilized to test the mediating effect of psychological flexibility on the relationship between illness perception and fear of recurrence, data was controlled for demographic covariates. The significance of the mediation effect was determined using the bootstrap method with 5000 samples. A two-tailed *p*-value less than 0.05 was considered statistically significant.

## 3. Results

### 3.1 Descriptive Statistics and Demographic Differences in Fear of Recurrence Among MI Patients

The mean score for fear of recurrence among MI patients in this study was 114.38 ± 28.23. Table 1 presented the differences in fear of recurrence across various demographic variables. Results showed that: gender, monthly income, marital status, alcohol consumption, NYHA classification and number of chronic diseases were significantly associated with fear of recurrence. Female patients exhibited significantly higher fear of recurrence scores compared to male patients (*p* < 0.001). Higher-income was associated with a lower fear of recurrence scores. Patients with a monthly income ≤714 USD had the highest fear of recurrence scores (*p* < 0.001), while those with a monthly income >1124 USD had the lowest scores. Patients who were unmarried or had other marital states demonstrated significantly higher fear of recurrence compared with married patients (*p* < 0.001). Patients who consumed alcohol had significantly higher fear of recurrence scores than those who did not (*p *= 0.027). Poorer cardiac function was associated with higher fear of recurrence scores. Patients with NYHA class I had the lowest fear of recurrence scores, while those with NYHA class IV had the highest scores (*p* < 0.001). Patients with two or more chronic diseases had significantly higher fear of recurrence scores compared to those without chronic diseases (*p* < 0.001).

**Table 1.** **Demographic differences in patients with myocardial infarction fear of recurrence (n = 466)**.

Variable		n (%)	Fear of recurrence, Mean ± SD	*p*
Age (years)				0.497
	≤50	88 (18.88)	110.82 ± 25.96	
	51∼60	190 (40.77)	115.22 ± 28.99	
	61∼70	126 (27.04)	116.37 ± 29.41	
	>70	62 (13.31)	112.79 ± 26.59	
Gender				<0.001
	Male	191 (40.99)	104.70 ± 27.03	
	Female	275 (59.01)	121.10 ± 27.12	
Residence				0.872
	Urban	340 (72.96)	114.25 ± 27.93	
	Rural	126 (27.04)	114.72 ± 29.14	
Education level				0.378
	Specialties	252 (54.08)	114.26 ± 29.01	
	Undergraduates	127 (27.25)	116.74 ± 28.68	
	Masters and above	87 (18.67)	111.26 ± 25.08	
Monthly income (USD)				<0.001
	≤714	154 (33.05)	133.10 ± 23.86	
	715∼1124	160 (34.33)	107.62 ± 21.61	
	>1124	152 (32.62)	102.51 ± 28.94	
Marital status				<0.001
	Unmarried			
	Other	39 (8.37)	128.36 ± 12.26	
	Married	427 (91.63)	113.10 ± 28.93	
Smoking				0.808
	No	278 (59.66)	114.64 ± 27.83	
	Yes	188 (40.34)	113.99 ± 28.89	
Alcohol				0.027
	No	241 (51.72)	111.58 ± 28.11	
	Yes	225 (48.28)	117.37 ± 28.11	
NYHA				<0.001
	I	66 (14.16)	93.41 ± 27.71	
	II	168 (36.05)	109.06 ± 25.20	
	III	158 (33.91)	123.80 ± 28.06	
	IV	74 (15.88)	125.01± 21.82	
MI type				0.537
	STEMI	201 (43.13)	115.30 ± 28.03	
	NSTEMI	265 (56.87)	113.67 ± 28.41	
Combine the number of chronic diseases				<0.001
	0	342 (73.39)	107.01 ± 22.67	
	1	85 (18.24)	123.26 ± 25.59	
	≥2	39 (8.37)	159.64 ± 30.43	

Note: SD, standard deviation; NYHA, New York Heart Association functional classification; MI, myocardial infarction; STEMI, ST-elevation myocardial infarction; NSTEMI, non-ST-elevation myocardial infarction.

### 3.2 Correlation Analysis of Fear of Recurrence, Illness Perception, and Psychological Flexibility

Table 2 presented the results of the correlation analysis among fear of recurrence, illness perception and psychological flexibility. Fear of recurrence was significantly positively correlated with illness perception (*r *= 0.724, *p* < 0.001), indicating that patients with stronger negative perceptions of their illness experienced higher levels of fear of recurrence. Fear of recurrence was significantly negatively correlated with psychological flexibility (*r *= –0.696, *p* < 0.001), suggesting that patients with higher levels of psychological flexibility reported lower levels of fear of recurrence. Illness perception and psychological capital were also significantly negatively correlated (*r *= –0.833, *p* < 0.001), demonstrating that more positive cognitive appraisals of the illness were associated with stronger psychological flexibility.

**Table 2.** **Description statistics and correlation analysis of each variable**.

Variables	Fear of recurrence		Psychological flexibility	
	*r*	*p*	*r*	*p*
Illness perception	0.724	<0.001	–0.833	<0.001
Psychological flexibility	–0.696	<0.001	-	-

### 3.3 Hierarchical Regression Analysis

To further investigate the influence of illness perception, psychological flexibility and other variables on fear of recurrence among MI patients, a hierarchical regression analysis was conducted (see Table 3). Illness perception emerged as a significant predictor of fear of recurrence (β = 0.930, *p* < 0.001), indicating that patients with stronger negative perceptions of their illness experienced higher levels of fear of recurrence. When psychological flexibility was added to the model, illness perception remained a significant predictor of fear of recurrence (β = 0.603, *p* < 0.001). However, the negative impact of psychological flexibility also became significant (β = –0.349, *p* < 0.001), suggesting that psychological flexibility partially mediated the relationship between illness perception and fear of recurrence.

**Table 3.** **Summary of hierarchical regression analyses predicting fear of recurrence**.

Outcome variables	Predictor variables	*R* ^2^	β	*SE*	*p*
Fear of recurrence	Illness perception	0.701	0.930	0.058	<0.001
	Gender		7.336	1.495	<0.001
	Monthly Income		–5.044	0.972	<0.001
	Marital status		6.691	2.674	0.039
	NYHA		7.076	0.811	0.322
	Alcohol		2.237	1.456	0.921
	Combine the number of chronic diseases		13.277	1.268	<0.001
Psychological flexibility	Illness perception	0.699	–0.936	0.036	<0.001
	Gender		–0.723	0.922	0.880
	Monthly Income		0.941	0.599	0.077
	Marital status		–0.505	1.649	0.785
	NYHA		–0.613	0.500	0.961
	Alcohol		0.637	0.898	0.504
	Combine the number of chronic diseases		–1.539	0.782	0.067
Fear of recurrence	Illness perception	0.715	0.603	0.089	<0.001
	Psychological flexibility		–0.349	0.074	<0.001
	Gender		7.309	1.462	<0.001
	Monthly Income		–4.716	0.953	<0.001
	Marital status		6.515	2.615	0.040
	NYHA		6.862	0.795	0.316
	Alcohol		2.459	1.424	0.061
	Combine the number of chronic diseases		12.740	1.245	<0.001

Note: SE, standard error.

### 3.4 Mediation Analysis

Based on the results of the hierarchical regression analysis, the mediation effect of psychological flexibility on the relationship between illness perception and fear of recurrence was significant. Illness perception indirectly influenced fear of recurrence by reducing psychological flexibility (β = –0.349, *p* < 0.001). Further analysis revealed that psychological flexibility accounted for 35.1% of the total effect, indicating that it played a substantial role in mitigating the impact of negative illness perceptions on fear of recurrence. These findings suggest that enhancing psychological flexibility could significantly alleviate fear of recurrence in MI patients by mediating the adverse effects of negative illness perceptions (see Table 4).

**Table 4.** **Direct and indirect effects of social responsibility on recurrence**.

Path	β	*SE*	95% CI	Ratio of effect values
Total effect	0.929	0.058	[0.816, 1.043]	
Direct effect	0.603	0.089	[0.427, 0.778]	64.9%
Indirect effect	0.326	0.067	[0.197, 0.460]	35.1%

## 4. Discussion

### 4.1 Fear of Recurrence is Relatively Severe Among MI Patients

MI is a sudden cardiovascular disease and its recurrence often leads to rapid and severe physical consequences, potentially even death. Compared with other chronic diseases, MI recurrence has a higher acute mortality rate, which intensifies patient fear of recurrence. Zigmond *et al*. [40] found that MI patients, having experienced severe cardiovascular symptoms (such as chest pain and dyspnea) and emergency treatment, tend to directly associate recurrence with death, resulting in a significantly higher fear of recurrence scores. MI recurrence is often unpredictable and patients may suffer a recurrent MI without apparent symptoms or warning signs. This unpredictability exacerbates patients fear, as they cannot completely avoid recurrence through self-monitoring or preventive behaviors. Bunde & Martin [41], showed that MI patients often exhibit higher levels of helplessness and anxiety due to their lack of control over recurrence and that this sense of helplessness is a significant source of fear of recurrence. In contrast, although chronic diseases such as diabetes and hypertension can lead to serious complications, their recurrence or worsening of the condition is usually gradual and does not immediately threaten life. As a result, fear of recurrence scores among those chronic disease patients is lower. Katon *et al*. [42] found that fear of recurrence in diabetes patients stems more from concerns about long-term complications rather than direct life threats. De Ridder *et al*. [43] pointed out that diabetes patients can control the progression of their disease through behavioral changes and medication and this sense of control helps reduce fear of recurrence.

The current study found that female patients had significantly higher fear of recurrence compared to male patients. This result is consistent with previous research, as existing evidence has shown that women exhibit more intense psychological stress responses after experiencing cardiovascular events [44]. This may be related to women being more sensitive about their health status, having higher levels of anxiety and facing pressures from social roles [45].

The study also found that patients with lower monthly incomes had higher fear of recurrence. The impact of economic status on health has been widely studied and low-income patients often face greater financial pressures, which not only increases their life burden but also affects their mental health [46]. Financial stress makes patients more worried about the economic consequences of disease recurrence, such as treatment costs and loss of work capacity, thereby intensifying their fear of recurrence [47].

This study indicated that married patients had significantly lower fear of recurrence compared to unmarried or divorced patients. Marriage is considered an important form of social support that effectively alleviates the psychological burden caused by disease [48]. Marital relationships provide both emotional and financial support as well as practical help, all of which contribute to reducing fear of disease recurrence for patients.

The study found that as the NYHA classification increased, patient fear of recurrence also significantly increased. The NYHA classification reflects the severity of heart function, and the worse the function, the greater a patient’s fear of disease recurrence. Patients with severe conditions often require more frequent medical interventions, which may make them more focused on the negative consequences of the disease, thereby increasing fear of recurrence [49].

This study showed that patients with multiple chronic diseases had significantly higher fear of recurrence compared to patients with no chronic diseases or only one chronic disease. The presence of multiple comorbidities often increases the psychological burden on patients, especially when they are facing multiple health problems [50]. The cumulative effect of diseases can significantly exacerbate recurrence fears.

### 4.2 Negative Illness Perception Exacerbates Fear of Recurrence in MI Patients

This study found that the negative perceptions held by MI patients significantly increased their fear of disease recurrence. Negative illness perception refers to patient understanding and interpretation of a disease that presents negative, incorrect, or exaggerated characteristics, usually manifested as excessive worry about disease severity, distrust of treatment effectiveness and uncertainty about future health. According to Leventhal’s self-regulation model [13], perceptions of the disease directly influence an individuals emotional responses and behavioral choices. When a patient believes that the disease is uncontrollable and the future is unpredictable, their anxiety and fear emotions significantly increase. Ames *et al*. [51] further validated this model, pointing out that negative illness perceptions, such as exaggerated understanding of the condition, often exacerbate patient emotional distress, leading to higher anxiety and fear of recurrence. Secondly, negative illness perception may lead to lack of confidence in treatment for a patient, thereby increasing their worry about disease recurrence. Weinstein *et al*. [52] showed that if patients believe that the treatment effect is poor or that disease recurrence is inevitable, they often generate a stronger sense of helplessness. This cognitive helplessness directly leads to an increased fear of disease recurrence. Moreover, negative perceptions may also weaken self-efficacy and hinder a patient from adopting positive health behaviors, further increasing their perceived risk of recurrence. In clinical practice, improving illness perception can help a patient establish more positive and realistic perceptions of the disease, alleviate fear of recurrence, enhance self-efficacy and perceived social support and ultimately improve both their quality of life and mental health.

### 4.3 The Protective Role of Psychological Flexibility

This study found that psychological flexibility is negatively correlated with fear of recurrence in MI patients. Psychological flexibility refers to an individual’s ability to accept negative emotions and thoughts and flexibly adjust their behavior to achieve long-term goals even when facing emotional distress. Psychological flexibility plays a key role in helping MI patients reduce fear of recurrence, especially through mechanisms such as emotion regulation, cognitive defusion and behavioral adjustment to alleviate fear. Hayes *et al*. [21] noted that individuals with high psychological flexibility are better able to accept negative emotions and maintain psychological balance while adopting behaviors that align with their long-term health goals, such as adhering to treatment plans and healthy lifestyles. This ability to accept and take action can reduce patient anxiety about disease recurrence. Kashdan & Rottenberg [20] showed that psychological flexibility allows individuals to engage in positive health behaviors even when faced with negative cognitions brought about by a disease, rather than being trapped by anxiety and fear. This cognitive and behavioral defusion helps patients avoid being overly immersed in their fear of recurrence. Psychological flexibility also enhances patient ability to adapt to life changes brought about by a disease by helping them effectively regulate negative emotions. Fledderus *et al*. [53] showed that patients with high psychological flexibility more quickly recover emotional balance and deal with the possibility of recurrence without being overwhelmed by fear when facing health crises. Clinical interventions should focus on cultivating psychological flexibility, helping patients reduce fear of recurrence and improve rehabilitation outcomes through psychological interventions and supportive therapy.

### 4.4 Psychological Flexibility Mediates the Negative Impact of Illness Perception on Fear of Recurrence

The study also found that psychological flexibility plays a mediating role between illness perception and fear of recurrence. That is, psychological flexibility alleviates the negative perceptions of MI patients to their disease, thereby reducing fear of recurrence. Psychological flexibility allows individuals to detach from their thoughts, viewing them as transient mental events rather than literal truths. For MI patients, this helps them break free from negative illness perceptions and have a more balanced view of their recovery, thus reducing fear of recurrence [54]. Acceptance is a key component of psychological flexibility, which involves allowing oneself to have various experiences without trying to change their frequency or form. By learning to accept physical discomfort and fear, MI patients can directly face their inner experiences, preventing avoidance behaviors that may exacerbate anxiety [23]. Simultaneously, psychological flexibility encourages individuals to clarify their values and take actions consistent with them. For MI patients, focusing on personally meaningful activities such as family, work and hobbies can shift their attention away from the disease, enhance their sense of fulfillment and thus alleviate disease-related fears [22].

## 5. Strengths and Limitations

This study provides valuable insights into the mediating role of psychological flexibility on the relationship between disease cognition and fear of recurrence in MI patients. A key strength lies in its focus on psychological flexibility, a construct increasingly recognized for its role in emotional regulation and behavior adjustment in the context of chronic illness. The findings offer a foundation for developing psychological interventions that enhance patient ability to accept negative emotions and maintain health-promoting behaviors despite recurring fears of illness.

Nevertheless, several limitations should be acknowledged. First, the sample limitations—including the regional focus and single-center design—may limit the generalizability of the results obtained to broader populations. Future research should consider multi-center studies with more diverse samples. Second, the cross-sectional design precludes any determination of causality between psychological flexibility and fear of recurrence. Longitudinal studies are needed to confirm the long-term effects of psychological flexibility. Third, reliance on self-reported measures for assessing fear of recurrence, disease cognition and psychological flexibility introduces the possibility of subjective bias, as participants may unintentionally misreport their psychological states.

Future research should explore the alternative model through longitudinal or experimental designs to disentangle the temporal and causal relationships between illness perception, psychological flexibility, and fear of recurrence. Additionally, further research is needed to explore the role of psychological flexibility in other chronic disease populations and to investigate novel intervention strategies aimed at reducing fear of recurrence by improving psychological flexibility.

## 6. Conclusion

This study explored the mediating role of psychological flexibility in the relationship between disease cognition and fear of recurrence among MI patients. Psychological flexibility, which enables patients to accept negative emotions and adjust their behavior in the face of illness, plays a crucial role in reshaping patient perception of their disease and reduces anxiety about recurrence. By enhancing psychological flexibility through interventions such as acceptance-based therapies, patients can better regulate their emotions, develop adaptive coping strategies and focus on long-term health goals, thereby lowering their fear of recurrence. Future clinical practice should incorporate psychological flexibility training to improve both the emotional well-being and recovery outcomes of MI patients.

## Data Availability

The data can be obtained with the consent of the author.
